# Progranulin inhibits autophagy to facilitate intracellular colonization of *Helicobacter pylori* through the PGRN/mTOR/DCN axis in gastric epithelial cells

**DOI:** 10.3389/fcimb.2024.1425367

**Published:** 2024-07-31

**Authors:** Linlin Liu, Miao Xiang, Jiaqi Zhou, Zongjiao Ren, Wenjing Shi, Xianhong Du, Xiaoyan Fu, Panpan Li, Hongyan Wang

**Affiliations:** ^1^ Key Laboratory of Immune Microenvironment and Inflammatory Disease Research in Universities of Shandong Province, School of Basic Medical Sciences, Shandong Second Medical University, Weifang, China; ^2^ Department of Pathogenic Biology, School of Basic Medical Sciences, Shandong Second Medical University, Weifang, China; ^3^ Health Toxicology Laboratory, School of Public Health, Shandong Second Medical University, Weifang, China; ^4^ School Hospital, Shandong Second Medical University, Weifang, China

**Keywords:** *Helicobacter pylori*, autophagy, PGRN, intracellular colonization, DCN

## Abstract

*Helicobacter pylori* (*H. pylori*) infection is the primary risk factor for the progress of gastric diseases. The persistent stomach colonization of *H. pylori* is closely associated with the development of gastritis and malignancies. Although the involvement of progranulin (PGRN) in various cancer types has been well-documented, its functional role and underlying mechanisms in gastric cancer (GC) associated with *H. pylori* infection remain largely unknown. This report demonstrated that PGRN was up-regulated in GC and associated with poor prognosis, as determined through local and public database analysis. Additionally, *H. pylori* induced the up-regulation of PGRN in gastric epithelial cells both *in vitro* and *in vivo*. Functional studies have shown that PGRN promoted the intracellular colonization of *H. pylori*. Mechanistically, *H. pylori* infection induced autophagy, while PGRN inhibited autophagy to promote the intracellular colonization of *H. pylori*. Furthermore, PGRN suppressed *H. pylori*-induced autophagy by down-regulating decorin (DCN) through the mTOR pathway. In general, PGRN inhibited autophagy to facilitate intracellular colonization of *H. pylori* via the PGRN/mTOR/DCN axis. This study provides new insights into the molecular mechanisms underlying the progression of gastric diseases, suggesting PGRN as a potential therapeutic target and prognostic predictor for these disorders.

## 1 Introduction

*Helicobacter pylori* (*H. pylori*) is a Gram-negative microaerobic bacterium that colonizes the gastric mucosa. It initiates a chronic inflammatory response, which progresses through a multi-step gastric tumorigenesis cascade known as Correa’s cascade (Thrift et al., 2023). Upon infection, *H. pylori* establishes long-term colonization on the gastric mucosal surface and subsequently invades large cytoplasmic vacuoles, where it continues to survive and alter the molecular composition of the vacuole (Amieva et al., 2002). *H. pylori* internalization in gastric epithelial cells exhibit greater resistance to immune response and antibiotic treatment and played a major role in tumor progression (Sit et al., 2020). Therefore, despite acting as an extracellular pathogen, a comprehensive understanding of the intracellular colonization mechanisms of *H. pylori* is crucial for preventing and treating persistent infections.

Autophagy is an intracellular degradation process that cells use to degrade and recycle cellular components. The process involves the formation of special structures called autophagosomes, which engulf damaged organelles, proteins, other cellular components and invading pathogens. These autophagosomes then fuse with lysosomes, which contain enzymes that break down the contents of the autophagosome for recycling. Autophagy plays a crucial role in maintaining cellular homeostasis, eliminating damaged components (Miller and Thorburn, 2021; Debnath et al., 2023). Pathogenic microorganisms have evolved multiple strategies to regulate or impede autophagy, leading to sustained intracellular survival (Kim et al., 2019; Chidambaram et al., 2022; Zhou et al., 2023). Autophagy has been found to have a complex role in regulating the survival of *H. pylori*, although the specific mechanisms involved are not yet well understood.

Progranulin (PGRN), known as GEP, GP88, or PC cell-derived multifunctional growth factor, consists of 593 amino acid residues (Anakwe and Gerton, 1990). It is involved in various physiological processes, including cell development, cell cycle progression, vascular and tissue repair, as well as the growth of bone and cartilage (Chen Q. et al., 2022). Numerous studies have shown the abnormal expression of PGRN in various cancers, including gastric cancer (GC), where its overexpression is closely associated with tumor proliferation, aggressiveness and adverse prognostic outcomes (Chen S. et al., 2022; Zhao et al., 2020; Do et al., 2021; Purrahman et al., 2022). Our preceding investigation revealed that *H. pylori* infection upregulated PGRN expressions in gastric epithelial cells and activated related signaling pathways, thereby enhancing the proliferation and migration of gastric cancer cells (Wang et al., 2011). Therefore, we aim to further investigate the mechanism through which PGRN exerts its oncogenic role. It has been documented that PGRN inhibits autophagy, thereby reducing MHC class I (MHC I) expression, limiting CD8^+^ T cell infiltration, and promoting immune evasion in pancreatic ductal adenocarcinoma (Cheung et al., 2022). However, the interplay between PGRN and autophagy within gastric mucosal epithelial cells has not been investigated, and it remains unclear whether the upregulation of PGRN triggered by *H. pylori* infection influences autophagy, potentially facilitating the colonization of *H. pylori* in these cells.

To further elucidate the mechanism by which PGRN modulates autophagy and contributes to *H. pylori* colonization, we employed gene chip analysis and identified that decorin (DCN), as the primary target protein of PGRN, indicating its role in regulating *H. pylori* intracellular colonization. DCN, a small leucine-rich proteoglycan, is crucial in mediating a variety of cellular functions, including proliferation, differentiation, and inflammatory responses, by interacting with multiple growth factors and receptors (Schneider et al., 2021). Moreover, DCN functions as a tumor suppressor in GC, with its expression levels significantly associated with angiogenesis and patient prognosis, suggesting its potential as a therapeutic target for GC (Basak et al., 2021). Recent research has demonstrated that DCN can modulate the tumor microenvironment, thus inhibiting tumor progression by inducing autophagy and apoptosis (Wang et al., 2020; Liu et al., 2022). However, the precise role of DCN in PGRN-mediated autophagy warrants further investigation.

In this study, we elucidated the association between *H. pylori* intracellular colonization and autophagy, analyzing the impact of PGRN on the internalization of *H. pylori* and its mechanistic role in inhibiting autophagy via the PGRN/mTOR/DCN pathway.

## 2 Materials and methods

### 2.1 Cell culture and reagents

Human immortalized gastric epithelial cell line GES−1 and human GC cell lines BGC-823 were cultured in RPMI−1640 medium (Gibco, USA), supplemented with 10% newborn bovine serum (Gibco, USA) and maintained in a humidified atmosphere containing 5% CO_2_ at 37°C. The autophagy inhibitor 3-MA (Selleck, USA) and mTOR inhibitor Everolimus (Selleck, USA) were dissolved in dimethyl sulfoxide (DMSO, China) for use in experiments.

### 2.2 Bacterial culture

As previously described, the wild-type *H. pylori* strain 26695 and SS1 were maintained in our laboratory. Briefly, the *H. pylori* strains were cultured in Brucella broth containing 5% FBS at 37°C under microaerobic conditions. Gastric cells were cocultured with *H. pylori* 26695 as a concentration of different multiplicity of infection (MOI) for designated durations.

### 2.3 Clinical samples

Atrophic gastric specimens were collected from 43 patients undergoing gastroscopic examination at the Affiliated Hospital of Shandong Second Medical University. These samples included *H. pylori*-positive chronic gastritis (n=24) and *H. pylori*-negative controls (n=19). 16S rRNA test and ^13^C-urea breath test were used to demonstrate *H. pylori* infection status. Patients were regarded as being *H. pylori*-positive if two tests yielded positive results. None of the patients had taken nonsteroidal anti-inflammatory drugs, antibiotics or proton pump inhibitors in the four weeks prior to the study. Additionally, 35 samples of paraneoplastic tissues and 50 GC tissue specimens were collected. The GC tissue specimens included *H. pylori*-negative samples (n=11), *H. pylori*-positive samples (n=32), and 7 samples that were not tested with ^13^C-urea breath test. Informed consent was obtained from each patient before participation. Data on age, gender, and pertinent clinical history were collected for all subjects in accordance with the approval from the Ethics Committee at Shandong Second Medical University (2022YX045).

### 2.4 Animal models

Specific pathogen-free (SPF) C56BL/6 mice (female, four weeks old) were used for *H. pylori* SS1 infection *in vivo* and purchased from Beijing Huafukang Bio-technology Co (SCXK(Beijing)2019–0008). All mice were randomly divided into two groups: uninfected (n=6) and *H. pylori*-infected (n=6) based on “complete randomization” rules. Mice were inoculated via oral gavage with 2.2×10^8^ colony forming units of *H. pylori* strain SS1 every other day for one month. Gastric tissues were collected 24 weeks later for rapid urease testing to confirm successful colonization of *H. pylori*, followed by additional analyses. All procedures and animal experiments were approved by the Animal Care and Use Committee of Shandong Second Medical University (2021SDL555).

### 2.5 Database and gene enrichment analysis

The gene expression levels of PGRN in GC and the clinical information of GC patients were derived from the Gene Expression Profiling Interactive Analysis (GEPIA, http://gepia.cancer-pku.cn/) and The Cancer Genome Atlas Program (TCGA, https://www.cancer.gov/ccg/research/genome-sequencing/tcga) database. Analysis of PGRN malignant expression in GC microenvironment by CTD database (https://www.ctdbase.org/) and TISCH database (http://tisch.comp-genomics.org/home/). Based on the expression of PGRN in patients, the expression levels were classified as high or low using the median expression level as the standard, survival analysis for GC patients was performed using the Kaplan–Meier Plotter (https://kmplot.com/analysis/). Gene set enrichment analysis (GSEA, https://www.gsea-msigdb.org/gsea/index.jsp) algorithm was employed to identify pathways significantly enriched between PGRN low and high tumor cells.

### 2.6 Immunohistochemical staining

Gastric tissues were fixed in 4% paraformaldehyde, paraffin-embedded, sectioned, deparaffinized with xylene, and rehydrated in ethanol. Following standard protocols, immunohistochemistry was conducted. Color development was achieved using DAB chromogenic solution (ZSGB Biotech), with subsequent hematoxylin staining of nuclei. Observations were made with an orthogonal fluorescence microscope (Olympus, Japan). The AOD is displayed in the bar chart as the measurement metric, which were assessed by ImageJ and checked by the individual pathologist.

### 2.7 Plasmids and siRNAs

The plasmid overexpressing PGRN and its corresponding negative control vector (pcDNA3.1) were successfully constructed and preserved in the laboratory. The PGRN and DCN siRNAs were purchased from Genepharma along with control siRNA (siNC). The lipofectamine 2000 (Invitrogen, USA) facilitated the transfection of plasmids and siRNA into GC cells. All experimental procedures are performed according to the manufacturer’s instructions.

### 2.8 RNA extraction and quantitative real-time PCR

Trizol reagent (Invitrogen) was used to extract the total RNA from the GC cells or gastric tissues according to the manufacturer’s protocol. RNA was reverse-transcribed into cDNA with a ReverTra Ace qPCR RT Kit (Toyobo, Japan). The mRNA expression levels of PGRN, DCN and *H. pylori* 16S rRNA were determined by using SYBR Green kit (AG, China) and the QuantStudio™ 1 Plus System (Applied Biosystems, USA) according to the protocol of manufacturer. Calculation of target mRNA levels was based on the CT method and normalization to human β-actin expression. The primer sequences are as follows:

PGRN, forward-5’-GGACAGTACTGAAGACTCTG-3’, reverse-5’-GGATGGCAGCTTGTAATGTG-3’;
DCN, forward-5’-GACAACAACAAGCTTACCAGAG-3’, reverse-5’-TGAAAAGACTCACACCCGAATA-3’;
β-actin, forward-5’-AGTTGCGTTACACCCTTTCTTG-3’, reverse-5’-CACCTTCACCGTTCCAGTTTT-3’;
16S rRNA, forward-5’-TGAGTACAAGACCCGGGAAC, reverse-5’-CAGTTCGGATTGTAGGCTGC-3’.

### 2.9 Western blot

Cell and tissue proteins were lysed using RIPA buffer containing PMSF protease inhibitor (Solarbio, China). The total protein concentration was quantified using a NanoDrop Lite Spectrophotometer (Thermo Scientific, USA). Proteins were then resolved by SDS-PAGE using either 10% or 12% gels and subsequently transferred onto PVDF membranes. The membranes were blocked with 5% nonfat milk at room temperature for one hour before overnight incubation with specific primary antibodies at 4°C. Following primary antibody binding, the membranes were washed and incubated with HRP-conjugated secondary antibodies. HRP-linked anti-mouse IgG (7076) and anti-rabbit IgG (7074) antibodies were from Cell Signal Technology. The protein bands were visualized using an enhanced chemiluminescence (ECL) detection system (EMD Millipore, USA). The bar graph was the intensities of the corresponding bands, which were measured using ImageJ software and normalized to β-actin, dividing the target protein band intensity by the internal control protein band intensity. PGRN (sc-377036), β-actin (sc-47778) antibodies were from Santa Cruz Biotechnology. LC3B(ab192890), DCN (ab277636) antibodies were from Abcam, mTOR (A2445) and phospho-mTOR (AP0115) antibodies were from Abconal Biotechnology.

### 2.10 Transmission electron microscopy

Cells were collected, fixed in 2% paraformaldehyde, 0.1% glutaraldehyde and in 0.1 mol/L sodium cacodylate for 2 h, subsequently post-fixed in 1% osmium tetroxide (OsO_4_) for 1.5 h, and then stained with 3% aqueous uranyl acetate for 1 h. Following an additional wash, cells were dehydrated through a graded series of and embedded in Epon-Araldite resin (Canemco, Canada). Ultrathin sections (0.05 μm) were prepared using an ultramicrotome, counterstained with 0.3% lead citrate, and examined on HT7700 (Hitachi, Japan) electron microscopy.

### 2.11 Confocal laser scanning microscope

Cells were infected with RFP-LC3B-expressing lentivirus and selected using 2 µg/ml puromycin. Subsequently, they were co-transfected with either PGRN plasmids or siRNAs, followed by infection with *H. pylori* at MOI 100:1 for 12 h. The formation of LC3B autophagic puncta was then monitored through sequential scanning with confocal laser scanning microscopy (CLSM) (Leica, Germany).

### 2.12 Gentamicin protection assay

The GPA was performed as previous studies to assess the invasion of gastric cells by *H. pylori*. *H. pylori* was introduced to gastric cells seeded in 24-well plates with antibiotic-free RPMI 1640 medium at an MOI of 100:1 for various durations. Unattached bacteria were removed by washing with 1 mL of warm PBS per well, followed by gentamicin (100 μg/ml, G1272, Sigma) treatment for 1 h to eliminate extracellular bacteria. After incubation, cells were washed and lysed with 0.5% saponin (47036, Sigma) in PBS at 37°C for 15 min. The lysates were then serially diluted from 10^1^ to 10^3^ into Brucella broth solid medium to culture viable intracellular *H. pylori* and the colonies were counted after an incubation period of 3–5 days.

### 2.13 Statistical analysis

The experimental data were statistically processed using SPSS 24.0 statistical software. Experimental data of three independent repeats were shown as mean ± standard deviation (SD). Paired t-test was used to compare the means between two groups; one-way ANOVA and multi way ANOVA was used for multiple groups. Survival analysis was performed using Kaplan-Meier. Figures were created in GraphPad Prism 8.0. *P*<0.01 and *P*<0.05 were considered statistically significant.

## 3 Results

### 3.1 PGRN is up-regulated in GC and associated with poor prognosis

**Figure 1:**
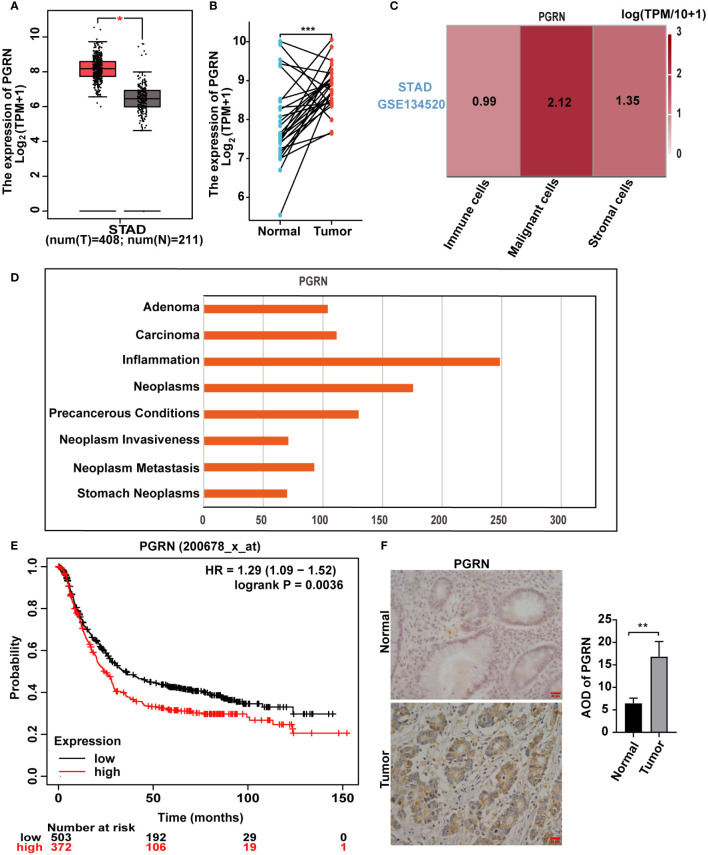
PGRN is up-regulated in GC and associated with poor prognosis. **(A)** Analysis of mRNA expression level of PGRN in GC tissues and non-tumor tissues according to TCGA database, with T representing GC tissue and N representing normal gastric mucosal epithelial tissue. **(B)** PGRN expression levels in GC and adjacent normal tissues across TCGA. The lines between the samples are one-to-one paired GC and para-cancerous tissue. **(C)** TISCH database analysis of PGRN expression in the microenvironment of GC (STAD-GSE134520). The number means the expression levels of PGRN in different cell types. **(D)** CTD database analysis of PGRN expression in malignant phenotype. **(E)** Kaplan-Meier analysis of overall survival (OS) in GC patients based on PGRN expression level. **(F)** Immunohistochemistry staining of PGRN in human adjacent normal and GC tissues. Scale bars, 20 μm. (*P<0.05, **P<0.01, ***P<0.001).

PGRN plays a significant and pivotal role in the modulation and progression of tumorous growths (Dong et al., 2023). We firstly elucidated the relationship between PGRN and GC. Our findings revealed a marked upregulation of PGRN in GC specimens compared with normal gastric tissues based on TCGA database (**Figures 1A, B**). Furthermore, PGRN levels were significantly higher in malignant cells than in other cellular constituents of the GC microenvironment (**Figure 1C**). Notably, PGRN was highest in inflammation phenotype among the various malignant phenotypes studied (**Figure 1D**). Building on previous studies that linked increased PGRN with poor prognosis in certain cancers, Kaplan-Meier Plotter survival analysis also showed that higher PGRN expression is associated with poorer overall survival in GC patients (**Figure 1E**). To evaluate the association between PGRN expression and clinicopathological parameters, we analysed PGRN mRNA expression in TCGA database. The results showed that up-regulation of PGRN corresponded with advanced tumor/node/metastasis (TNM) stage and correlated with the increased tumor size and/or the extent of invasion into adjacent tissues (**Supplementary Table 1**). We further used clinical gastric tissues to prove the correlation between PGRN and GC, demonstrating higher expression of PGRN in GC compared to normal gastric tissues (**Figure 1F**). Collectively, these findings indicate that PGRN is up-regulated in GC and is indicative of poor prognosis.

### 3.2 *H. pylori* induces the up-regulation of PGRN in gastric epithelial cells

**Figure 2:**
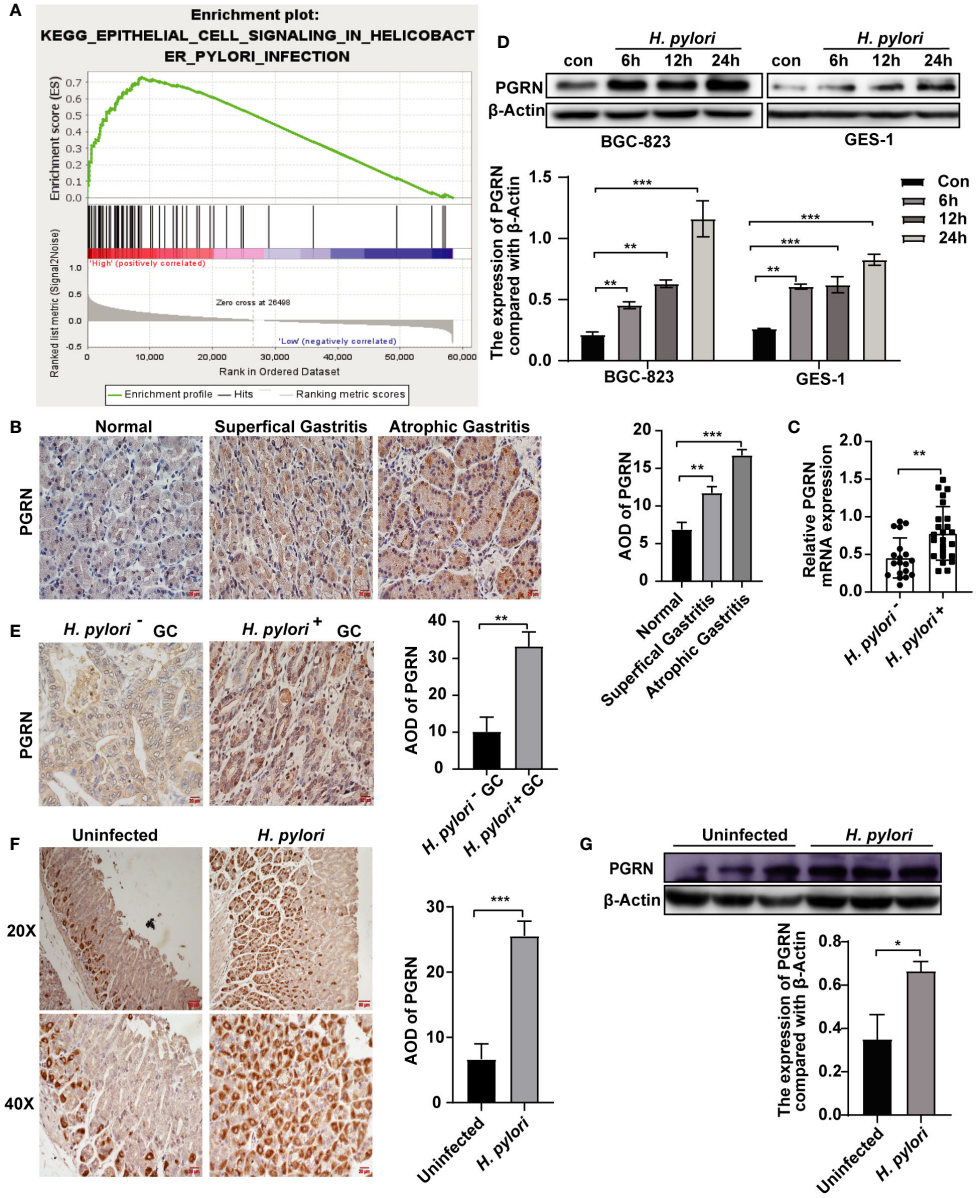
*H. pylori* induces the up-regulation of PGRN in gastric epithelial cells. **(A)** Enrichment plots of gene expression signatures for *H. pylori* infection according to PGRN mRNA expression in a GSEA analysis of TCGA. **(B)** Immunohistochemistry staining of PGRN in human normal, superficial gastritis and atrophic gastritis tissues. Scale bars, 20 μm. **(C)** qRT-PCR analysis of PGRN mRNA expression level in *H. pylori*-negative (n=19) and *H. pylori*-positive (n=24) chronic gastritis tissue samples. **(D)** Western blot of PGRN in in BGC-823 and GES-1 cells infected with *H. pylori* (MOI = 100) for 0, 6, 12 and 24 h. **(E)** Immunohistochemistry staining of PGRN in *H. pylori*-negative (n=11) and *H. pylori*-positive (n=32) GC tissues. Scale bars, 20 μm. **(F)** Immunohistochemistry staining of PGRN in containing *H. pylori*-negative and *H. pylori*- positive gastric tissues of mice. Scale bars, 50 μm (20X), 20 μm (40X). **(G)** Western blot of PGRN in *H. pylori*-negative and *H. pylori*-positive gastric tissues of mice. (*P<0.05, **P<0.01, ***P<0.001).

*H. pylori* infection is one of the major causes of chronic gastritis and GC. We further explore the association between *H. pylori* infection and PGRN. GSEA confirmed a significant association between PGRN mRNA expression and *H. pylori* infection (**Figure 2A**). To elucidate the clinical significance of PGRN in gastritis, we analyzed gastric tissues from patients at varying stages of gastritis and found a progressive increase in PGRN expression, ranging from normal gastric mucosa through superficial gastritis to atrophic gastritis (**Figure 2B**). Furthermore, comparative analysis revealed that PGRN mRNA expression in *H. pylori*-positive chronic gastritis tissues was higher than that in *H. pylori*-negative counterparts (**Figure 2C**). *In vitro* experiments demonstrated that *H. pylori* infection promoted PGRN expression in gastric epithelial cells (**Figure 2D**). This up-regulation was corroborated *in vivo* both in *H. pylori*-negative, *H. pylori*-positive GC tissues and a mouse model infected with *H. pylori* (**Figures 2E–G**). Therefore, it is evident that *H. pylori* triggers the up-regulation of PGRN in gastric epithelial cells.

### 3.3 PGRN promotes the intracellular colonization of *H. pylori*

**Figure 3:**
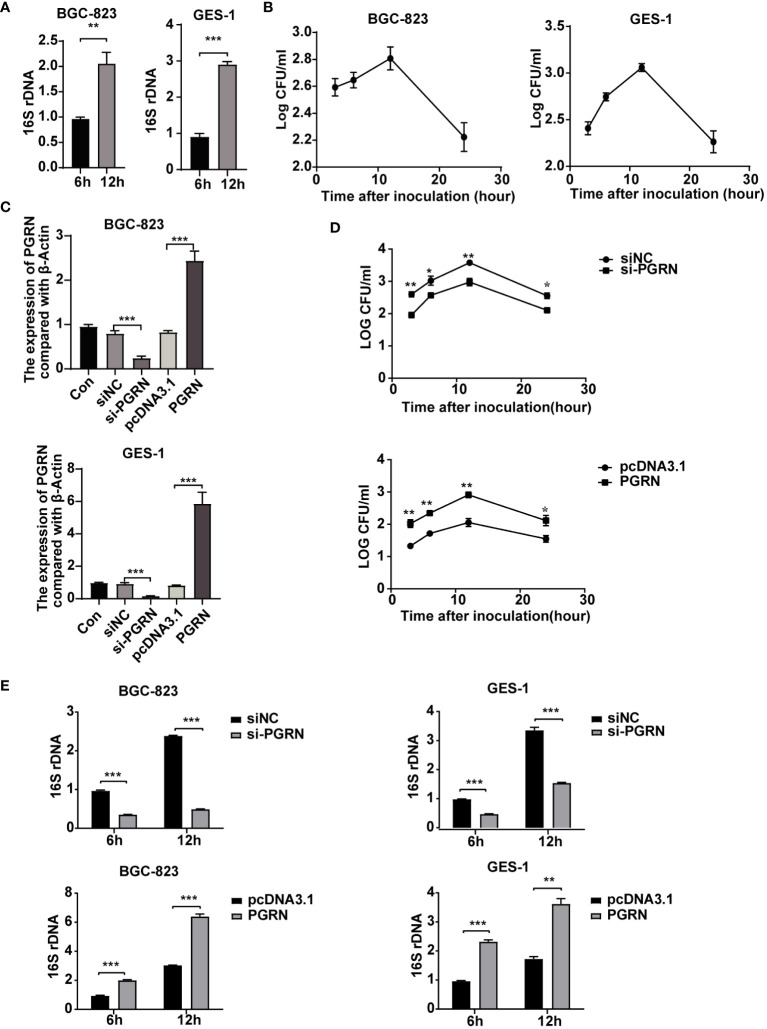
PGRN promotes the intracellular colonization of *H. pylori*. **(A)** qRT-PCR analysis of 16S rRNA in BGC-823 and GES-1 cells infected with *H. pylori* for 6 h, 12 h (n=3). **(B)** CFU analysis in BGC-823 and GES-1 cells infected with *H. pylori* (MOI = 100) for 3, 6, 12 and 24 h, then followed with gentamicin protection assay. **(C)** qRT-PCR analysis of PGRN mRNA expression level in BGC-823 and GES-1 cells transfected with PGRN siRNA or WT PGRN (n=3). **(D)** CFU analysis in BGC-823 cells transfected with PGRN siRNA or WT PGRN following *H. pylori* infection for indicated times, then followed with gentamicin protection assay (n=3). **(E)** qRT-PCR analysis of 16S rRNA levels in BGC-823 and GES-1 cells transfected with PGRN siRNA or WT PGRN following *H. pylori* infection for 6 h, 12 h (n=3). (*P<0.05, **P<0.01, ***P<0.001).

Although *H. pylori* was previously considered an extracellular bacterium, increasing evidence has shown that it can survive and even multiply inside gastric epithelial cells (Dubois and Borén, 2007). Therefore, our study aimed to investigate the impact of PGRN on the intracellular colonization of *H. pylori*. Utilizing *H. pylori* 16S rRNA and colony forming units assay followed by gentamicin treatment, we demonstrated the colonization of *H. pylori* within gastric epithelial cells (**Figures 3A, B**). Furthermore, overexpression of PGRN promoted the intracellular colonization of *H. pylori* into gastric epithelial cells, whereas knockdown of PGRN got the opposite effect (**Figures 3C–E**). These results indicate that PGRN promotes the intracellular colonization of *H. pylori*.

### 3.4 PGRN inhibits autophagy to promote intracellular colonization of *H. pylori*

Autophagy is involved in multiple biological processes regulated by *H. pylori* infection (Yang et al., 2022). To ascertain the effect of *H. pylori* on autophagy within gastric epithelial cells, we investigated the expression of LC3BI/II and the formation of RFP-LC3B puncta, a reliable autophagy marker, following *H. pylori* infection. Our findings showed that *H. pylori* infection promoted LC3B expression and RFP-LC3B puncta formation (**Supplementary Figures 1A, B**). These results indicate that *H. pylori* infection induces autophagy in gastric epithelial cells.

**Figure 4:**
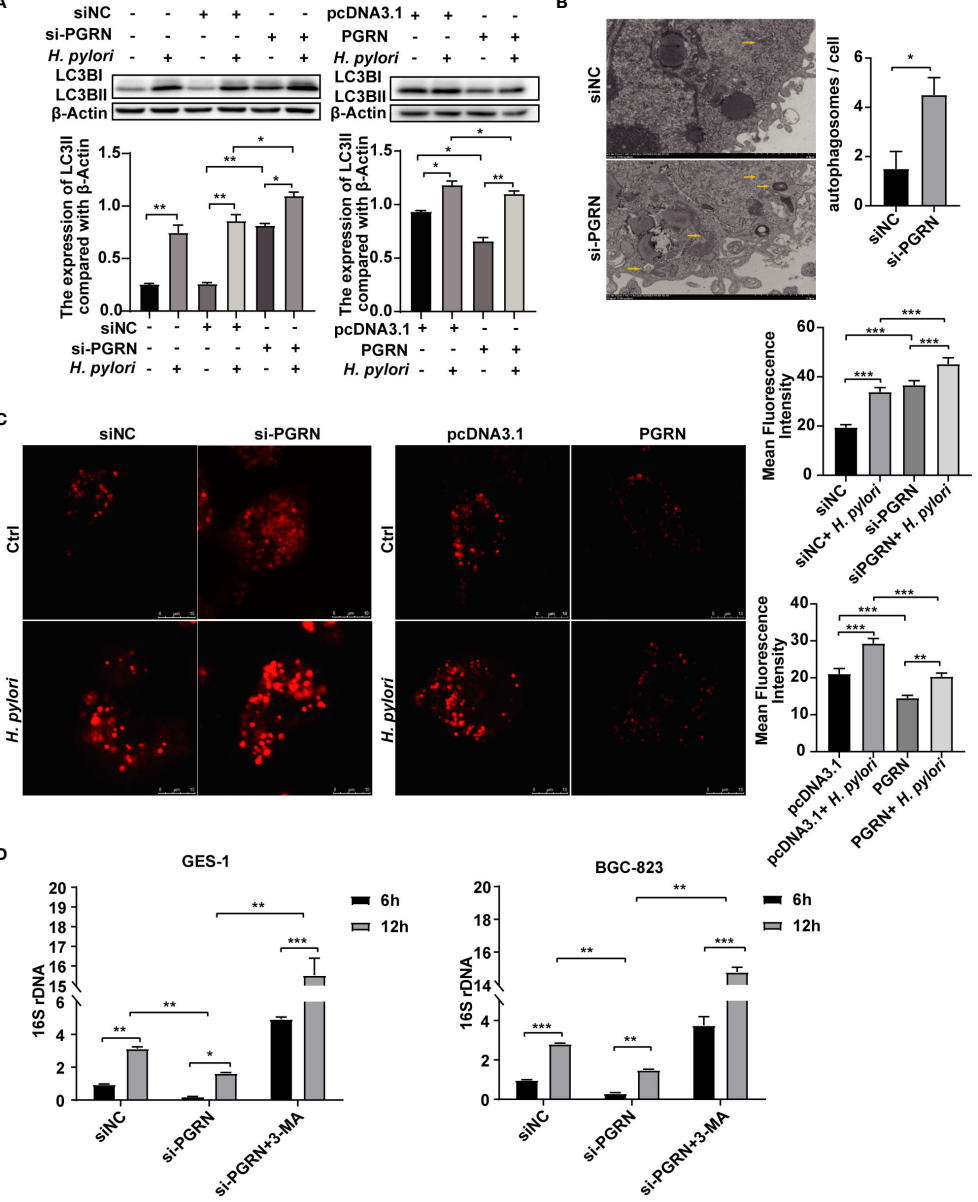
PGRN inhibits autophagy to promote intracellular colonization of *H. pylori.* **(A)** Western blot of LC3B in BGC-823 cells transfected with PGRN siRNA or WT PGRN following *H. pylori* infection for 12 h. **(B)** TEM images of autophagosomes in BGC-823 cells transfected with PGRN siRNA. Scale bars, 2 μm. **(C)** CLSM images of LC3B autophagy puncta in BGC-823 cells with stably expressing RFP-LC3B transfected with PGRN siRNA or WT PGRN following *H. pylori* infection. Scale bars, 10 μm. **(D)** qRT-PCR analysis of 16S rRNA levels in BGC-823 and GES-1 cells transfected with PGRN siRNA or WT PGRN following *H. pylori* infection for 12 h (MOI=100:1), with or without a pretreatment of the autophagy inhibitor 3-MA (10 nM, 2 h) (n=3). (*P<0.05, **P<0.01, ***P<0.001).

In order to investigate the underlying mechanism of PGRN in facilitating intracellular colonization of *H. pylori*, we employed gene microarray high pathway screening to examine the functions of PGRN involved in the regulation, revealing a close association between PGRN and the autophagic process in gastric epithelial cells (**Supplementary Figure 1C**). Meanwhile, GSEA highlighted the involvement of PGRN in regulating lysosome-associated pathways and cellular endocytosis (**Supplementary Figures 1D, E**), indicating a potential mechanism through which PGRN may influence *H. pylori*-induced autophagy. Furthermore, we found that down-regulation of PGRN significantly increased the expression of LC3B, while overexpression of PGRN had the opposite effects (**Figure 4A**). Using TEM, we also observed a significant increase in autophagosome formation in gastric epithelial cells following PGRN interference (**Figure 4B**). CLSM analysis further confirmed the difference in LC3B autophagy puncta formation corresponding with PGRN expression (**Figure 4C**). More importantly, the inhibition of 16S rRNA of *H. pylori* due to PGRN knockdown could be rescued in the cells pretreated with 3-MA (**Figure 4D**). Taken together, we confirm that PGRN promotes *H. pylori* colonization in gastric epithelial cells by inhibiting cellular autophagy.

### 3.5 PGRN suppresses *H. pylori*-induced autophagy via down-regulation of DCN

**Figure 5:**
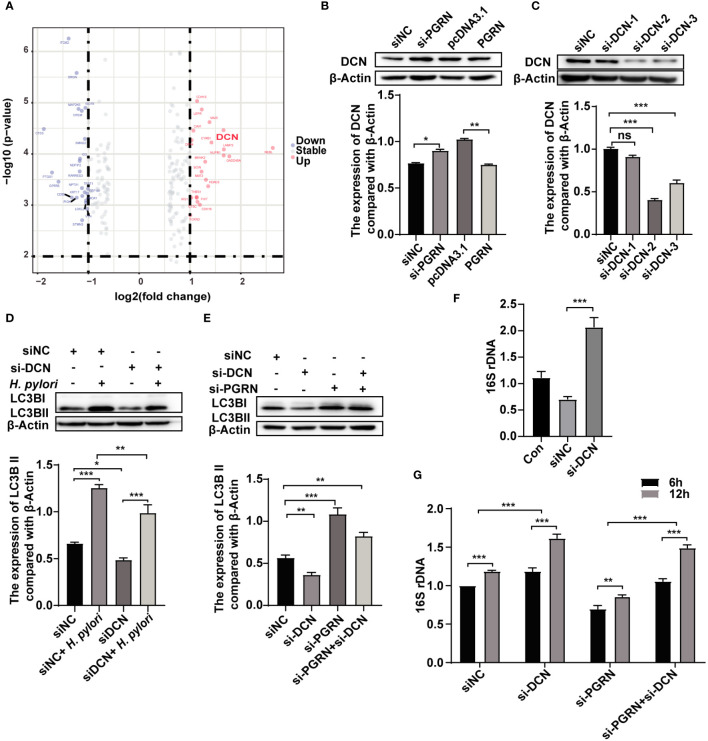
PGRN suppresses *H. pylori*-induced autophagy via down-regulation of DCN. **(A)** Gene microarray analysis of PGRN downstream target genes shown as volcano plots. **(B)** Western blot of DCN in BGC-823 cells transfected with WT PGRN and PGRN siRNA. **(C)** Western blot of DCN in BGC-823 cells transfected with DCN siRNA. **(D)** Western blot of LC3B in BGC-823 cells transfected with DCN siRNA following *H. pylori* infection for 12 h. **(E)** Western blot of LC3B in BGC-823 cells transfected with PGRN or/and DCN siRNA. **(F)** qRT-PCR analysis of 16S rRNA levels in BGC-823 cells transfected with DCN siRNA following *H. pylori* infection for 12 h (n=3). **(G)** qRT-PCR analysis of 16S rRNA levels in BGC-823 cells transfected with DCN siRNA, PGRN siRNA following *H. pylori* infection for 6, 12 h (n=3). (*P<0.05, **P<0.01, ***P<0.001, ns, not significant).

To investigate the role of PGRN in regulating autophagy, a gene microarray was conducted to predict the interaction proteins of PGRN, and several genes were identified as a potential PGRN-associated protein (**Figure 5A**; **Supplementary Table 2**). Subsequent validation experiments confirmed that PGRN could negatively regulate DCN expression (**Figure 5B**). To confirm the effects of DCN on autophagy in gastric epithelial cells, we detected LC3B expression after down-regulation of DCN. Results showed that DCN knockdown inhibited autophagy induced by *H. pylori* infection (**Figures 5C, D**). Additionally, down-regulation of DCN rescued the level of LC3B, which was increased by PGRN knockdown (**Figure 5E**). Subsequently, the effect of DCN on intracellular bacterial colonization was investigated, showing that down-regulation of DCN enhanced the intracellular colonization of *H. pylori* (**Figure 5F**). Furthermore, co-transfection with si-PGRN and si-DCN attenuated the reduction of intracellular *H. pylori* colonization initially induced by PGRN knockdown (**Figure 5G**). These findings indicate that PGRN inhibits *H. pylori*-induced autophagy via down-regulation of DCN.

### 3.6 PGRN down-regulates DCN by inhibiting the mTOR pathway

**Figure 6:**
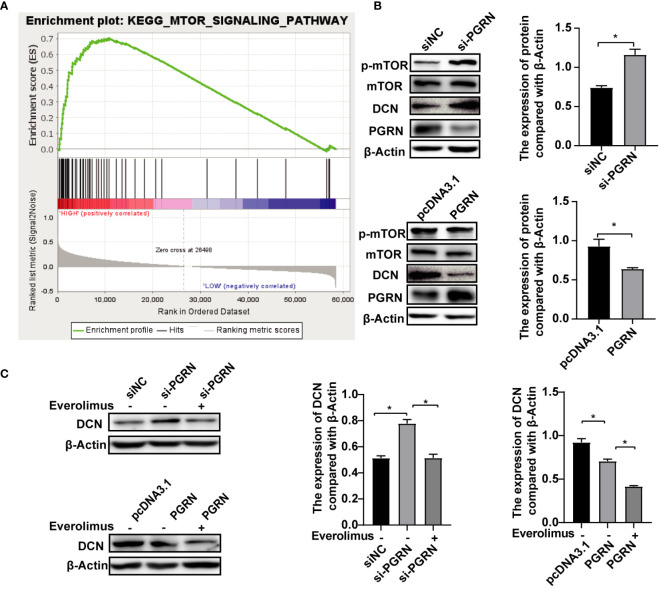
PGRN down-regulates DCN by inhibiting the mTOR pathway. **(A)** GSEA analyzed the involvement of PGRN in the mTOR pathway. **(B)** Western blot of mTOR signaling in BGC-823 cells transfected with PGRN siRNA or WT PGRN. **(C)** Western blot of DCN in BGC-823 cells transfected with PGRN siRNA or WT PGRN, then treated with Everolious (10 nM) for 2 h. (*P<0.05).

The biogenesis of autophagic vesicles is intricately linked to the regulation by upstream pathways, including the mechanistic target of the rapamycin (mTOR) pathway (Pei et al., 2023). GSEA analysis revealed the involvement of PGRN in modulating the mTOR pathway (**Figure 6A**), suggesting that PGRN may regulate autophagy by influencing DCN expression through the mTOR signaling pathway. Upon the siRNA-mediated down-regulation of PGRN, an increase in phosphorylated mTOR (p-mTOR) levels was observed, with a corresponding alteration following PGRN overexpression (**Figure 6B**). Furthermore, up-regulation of DCN by si-PGRN was rescued via inhibiting mTOR (**Figure 6C**). Taken together, we confirmed that PGRN down-regulated DCN by inhibiting the mTOR pathway. And these findings highlight the crucial role of the mTOR pathway in mediating the effects of PGRN on DCN expression, autophagy and intracellular colonization.

## 4 Discussion

*H. pylori*, a significant risk factor closely associated with gastric carcinogenesis, has become a focal point of current research due to the urgent need to improve diagnostic and therapeutic strategies (Malfertheiner et al., 2023). Once individuals acquire *H. pylori* infection, the pathogen can persists in the gastric mucosa for a lifetime if left untreated. Although *H. pylori* is considered an extracellular pathogen, it also has the ability to invade and colonize host cells, thereby evading clearance by the immune system and causing long-term infection. Zhang discovered that *H. pylori* infection can reduce the sensitivity of Toll-like receptors 6 (TLR6) and down-regulate the expression of inflammatory factors, leading to persistent infection (Zhang et al., 2024). Our study also found that *H. pylori* is capable of intracellular invasion, colonization, and survival, with this ability increasing over time, as shown by intracellular 16S rRNA detection and colony formation after gentamicin treatment. However, the mechanisms underlying *H. pylori*-induced intracellular colonization remain unclear.

Autophagy is a cellular “cleanup” process that cells use to degrade and recycle unnecessary or dysfunctional cellular components. It plays a crucial role in maintaining cellular homeostasis, removing damaged organelles, clearing protein aggregates, and combating invading pathogens like bacteria, parasites, and viruses. However, pathogens can evade host cell elimination through various mechanisms, resulting in persistent bacterial infections. Studies has shown that the heparin-binding hemagglutinin (HBHA) of *Mycobacterium tuberculosis* inhibited autophagy in macrophages via Toll-like receptor 4, thereby promoting intracellular survival (Zheng et al., 2024). Similarly, Uropathogenic *Escherichia coli* PldA suppressed pre-autophagosomal structures, decreasing lysosomal cytotoxicity and escaping host immunity (Li et al., 2024). Furthermore, *H. pylori* also developed highly evolved mechanisms to escape autophagic degradation, manipulate autophagic pathways, and reconstruct autophagosomal compartments for survival in gastric cells (Capurro et al., 2020). Therefore, a key mechanism for *H. pylori* to evade host immune responses and maintain infection involves inhibiting autophagy and facilitating intracellular colonization in gastric epithelial cells. In our study, we discovered that *H. pylori* infection promoted the formation of LC3B autophagy puncta and up-regulated the expression of the autophagy-associated protein LC3B, while inhibiting autophagy using 3-MA resulted in increased intracellular bacterial loading and survival. However, the mechanism by which *H. pylori* regulates autophagy are complex and requires further study.

PGRN is a growth factor that plays a crucial role in various biological processes, including inflammation, wound healing, neurodegeneration and cancer development and progression. It has been reported that PGRN modulates PD-1 expression in tumor-associated macrophages (TAMs), promotes CD8^+^ T cell rejection, and induces immune escape from breast cancer (Fang et al., 2021). Our previous research indicated that *H. pylori* can upregulate the expression of PGRN in gastric mucosal epithelial cells through the p38 MAPK and MEK1/2 signaling pathways. The elevated expression of PGRN can subsequently modulate G2/M stage and CDK4 to promote cell cycle progression, leading to the proliferation and migration of gastric cancer cells (Wang et al., 2011; Yang et al., 2017; Ren et al., 2022). While the intricate mechanisms by which PGRN regulate cell cycle and promote proliferation requires further study. Additionally, several studies have demonstrated that PGRN possesses pro-lysosomal functions, such as regulating protease activity, promoting lysosomal acidification, and facilitating protein trafficking (Tanaka et al., 2017; Zhou et al., 2017a, Zhou et al., 2017b). Therefore, it is worth investigating how *H. pylori*-induced upregulation of PGRN expression promotes lysosomal function in gastric mucosal epithelial cells. In this study, we present evidence that *H. pylori*-mediated PGRN induction, both *in vitro* and *in vivo*, suppresses autophagy, thus promoting the intracellular colonization and survival of *H. pylori* in gastric epithelial cells. Therefore, our findings may provide a novel mechanism that high expression of PGRN can inhibit autophagy in gastric mucosal cells, suppress pro-lysosomal functions, leading to the failure in eliminating intracellular *H. pylori*, resulting in persistent infection and proliferation of *H. pylori* in the host cells.

To elucidate the molecular mechanisms underlying PGRN function, we employed high-throughput gene chip screening and bioinformatic analyses, which identified DCN as a probable functional target. Our findings demonstrated that DCN expression is up-regulated in PGRN-knockdown gastric epithelial cells. As a matrix-derived proteoglycan, DCN functions as a partial agonist for a variety of biological activities, directly interacting with a wide range of receptor tyrosine kinases (RTKs) and activating autophagy (Neill et al., 2017). Our data indicated that *H. pylori* infection triggered autophagy in gastric epithelial cells, while downregulation of DCN expression curtailed autophagy thus facilitating intracellular *H. pylori* colonization. Meanwhile, PGRN-mediated cellular autophagy and intracellular colonization were rescued by DCN. The autophagy inhibited by DCN downregulation may result from reduced interactions with PTKs, which will be further investigated in subsequent studies. Additionally, PGRN is significantly enriched in the mTOR pathway. The mTOR complex plays a critical role not only in gene transcription regulation and tumor metabolism but also in the regulation of autophagy and apoptosis. Our study shows that PGRN can inhibit mTOR phosphorylation to down-regulate DCN expression. Chen et al. indicated that Nobiletin can inhibit the protective effect of autophagy in GC cells through the PI3K/Akt/mTOR pathway (Chen et al., 2024). Additionally, Wang et al. proposed that Vitamin D3 promotes autophagy in GC cells by mediating AMPK/mTOR pathway (Wang et al., 2024). Therefore, the upregulated PGRN by *H. pylori* may inhibit mTOR phosphorylation through the regulation of PI3K/AKT and AMPK. Additional experiments using the mTOR inhibitor Everolimus confirmed that PGRN impeded *H. pylori*-induced autophagy in gastric epithelial cells by modulating DCN expression through the mTOR pathway.

**Figure 7:**
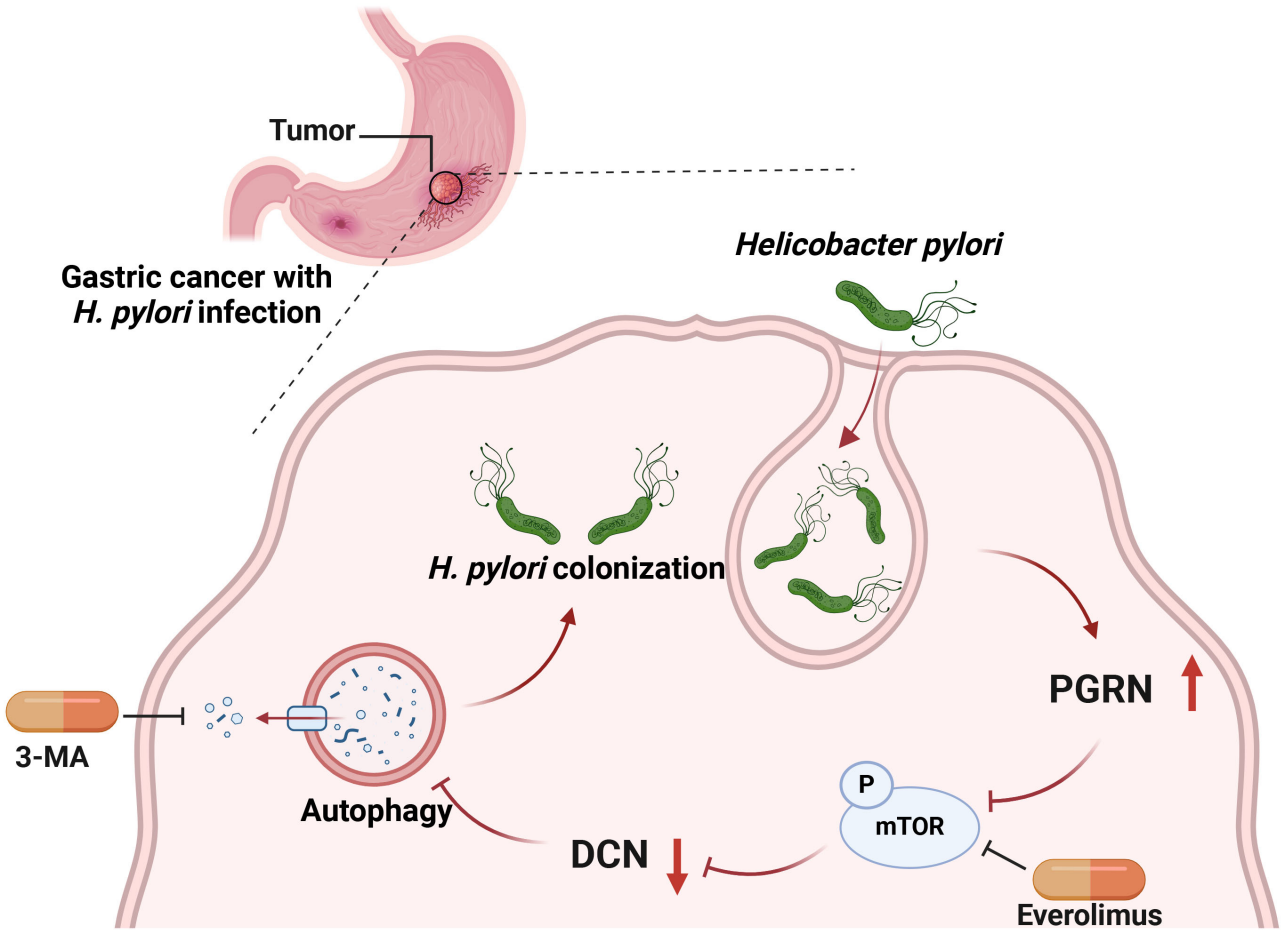
Working model for PGRN regulate autophagy and intracellular colonization in GC.

In conclusion, *H. pylori*, previously regarded as an extracellular bacterium, can colonize and even multiply inside gastric epithelial cells. Our study revealed that *H. pylori* infection induces upregulation of PGRN, which in turn facilitates the intracellular colonization of *H. pylori* by attenuating cellular autophagy. This suppression occurs via the downregulation of DCN, mediated by the inhibition of the mTOR pathway (**Figure 7**). Our findings provide a novel mechanism by which PGRN enhances *H. pylori* colonization and promotes proliferation of gastric epithelial cells. Targeting PGRN in *H. pylori* infection and GC may offer new opportunities for treatment strategies in the future.

## Data availability statement

The datasets presented in this study can be found in online repositories. The name of the repository and accession number can be found below: https://www.ncbi.nlm.nih.gov/geo/, GSE266076.

## Ethics statement

The studies involving humans were approved by the Ethics Committee at Shandong Second Medical University (2022YX045). The studies were conducted in accordance with the local legislation and institutional requirements. The participants provided their written informed consent to participate in this study. The animal study was approved by the Animal Care and Use Committee of Shandong Second Medical University (2021SDL555). The study was conducted in accordance with the local legislation and institutional requirements. Written informed consent was obtained from the individual(s) for the publication of any potentially identifiable images or data included in this article.

## Author contributions

LL: Writing – original draft, Project administration, Methodology, Investigation, Software. MX: Writing – original draft, Methodology, Investigation, Software, Project administration. JZ: Writing – original draft, Investigation, Project administration, Software, Methodology. ZR: Writing – original draft, Investigation, Methodology, Project administration, Software. WS: Writing – original draft, Funding acquisition, Methodology. XD: Writing – original draft, Methodology. XF: Writing – original draft, Methodology. PL: Writing – original draft, Writing – review & editing, Funding acquisition, Project administration, Validation, Formal analysis, Supervision, Software, Investigation, Methodology, Visualization, Resources. HW: Writing – original draft, Writing – review & editing, Supervision, Project administration, Validation, Methodology, Investigation, Formal analysis, Funding acquisition, Visualization, Resources, Software.
