# Supplementary material for: Progranulin inhibits autophagy to facilitate intracellular colonization of Helicobacter pylori through the PGRN/mTOR/DCN axis in gastric epithelial cells
Source: Front Cell Infect Microbiol. 2024 Jul 31;14:1425367. doi: 10.3389/fcimb.2024.1425367 (PMC11322814; doi:10.3389/fcimb.2024.1425367)
Supplement: Supplementary file 1 [file DataSheet_1.docx]

Supplementary Material

# Supplementary Figures

**Supplementary Figure 1.** ***H. pylori* induces autophagy in gastric epithelial cells.** (A) Western blot of LC3B in BGC-823 and GES-1 cells infected with *H. pylori* for 0, 6, 12, 24 h (MOI=100). (B) Fluorescence microscopy images of LC3B in BGC-823 and GES-1cells which stably expressed RFP-LC3 infected with *H. pylori* for 12 h. Scale bars, 10 µm. (C) Gene microarray analyze the function of PGRN involved in regulation. (D) GSEA analysis of PGRN enrichment in the lysosome pathway. (E) GSEA analysis of PGRN enrichment in the endocytosis pathway. **P<0.01; ***P<0.001

# Supplementary Tables

# Supplementary Table 1. Clinical correlation between PGRN mRNA expression and clinical and pathological

**Supplementary Table 2. Potential PGRN-associated proteins of gene microarray**
