# Supplementary figures and images for: Progranulin inhibits autophagy to facilitate intracellular colonization of Helicobacter pylori through the PGRN/mTOR/DCN axis in gastric epithelial cells
Source: Front Cell Infect Microbiol. 2024 Jul 31;14:1425367. doi: 10.3389/fcimb.2024.1425367 (PMC11322814; doi:10.3389/fcimb.2024.1425367)

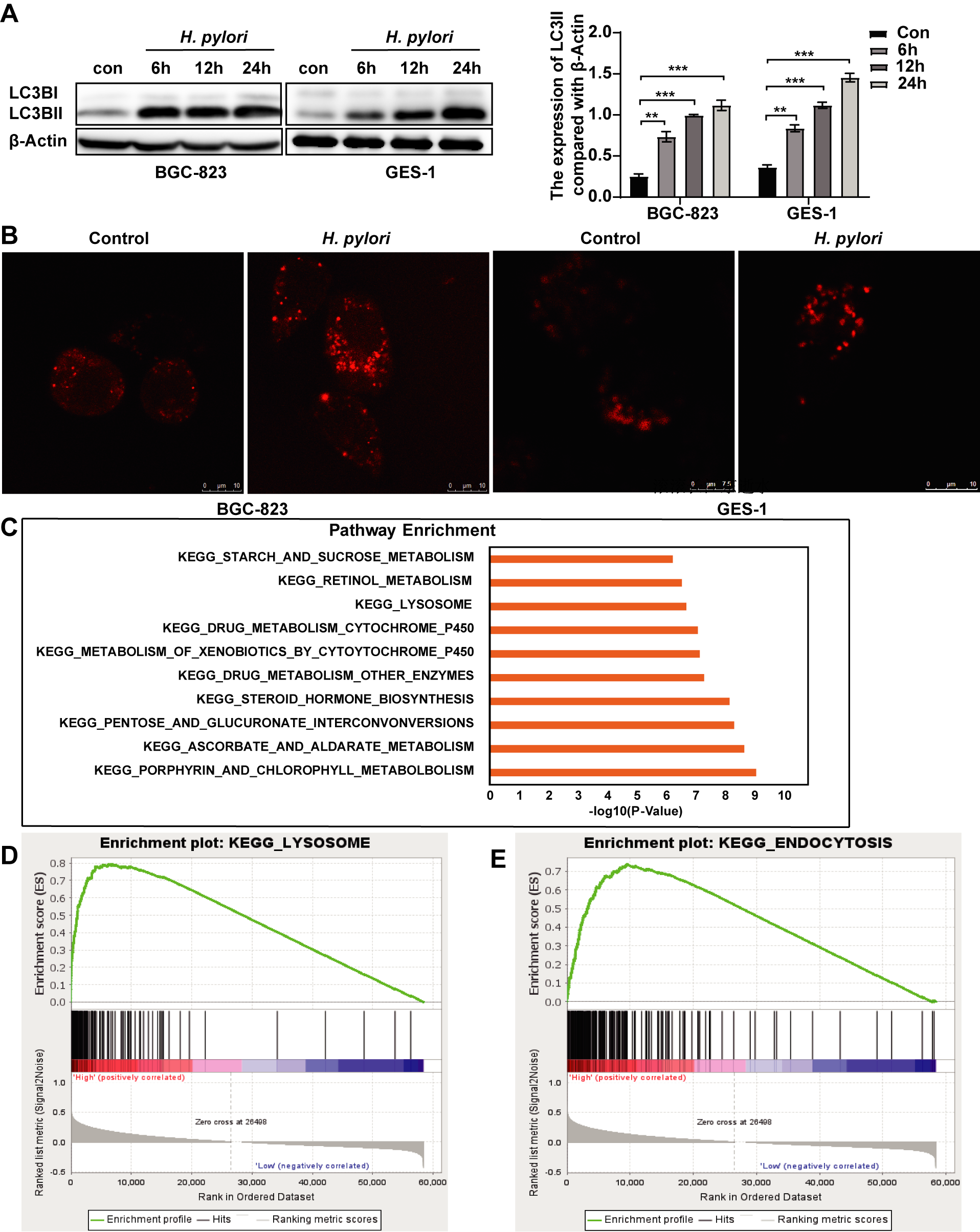

Supplement: Supplementary file 2 [file Image_1.tif]
