# Supplementary material for: Progranulin inhibits autophagy to facilitate intracellular colonization of Helicobacter pylori through the PGRN/mTOR/DCN axis in gastric epithelial cells
Source: Front Cell Infect Microbiol. 2024 Jul 31;14:1425367. doi: 10.3389/fcimb.2024.1425367 (PMC11322814; doi:10.3389/fcimb.2024.1425367)
Supplement: Supplementary file 3 [file Table_1.docx]

Supplementary Material

# Supplementary Table 1. Clinical correlation between PGRN mRNA expression and clinical and pathological

| **Clinical**  **Characteristics** | **PGRN mRNA expression** | | ***P valve*** |
| --- | --- | --- | --- |
|  | **Low (n=188)** | **High (n=189)** |  |
| **Gender** |  |  |  |
| Male | 120 | 123 | 0.214 |
| Female | 68 | 66 |  |
| **Age(years)** |  |  |  |
| ≤70 | 121 | 108 | 0.733 |
| >70 | 67 | 81 |  |
| **Pathological TNM stage** |  |  |  |
| Ⅰ | 27 | 30 | 0.015 |
| Ⅱ | 65 | 55 |  |
| Ⅲ | 83 | 82 |  |
| Ⅳ | 15 | 22 |  |
| **Pathological T stage** |  |  |  |
| 1 | 10 | 9 | 0.013 |
| 2 | 34 | 44 |  |
| 3 | 96 | 73 |  |
| 4 | 48 | 63 |  |
| **Pathological N&M stage** |  |  |  |
| 0 | 173 | 165 | 0.284 |
| 1 | 10 | 14 |  |
| X | 5 | 10 |  |
