# Supplementary material for: Progranulin inhibits autophagy to facilitate intracellular colonization of Helicobacter pylori through the PGRN/mTOR/DCN axis in gastric epithelial cells
Source: Front Cell Infect Microbiol. 2024 Jul 31;14:1425367. doi: 10.3389/fcimb.2024.1425367 (PMC11322814; doi:10.3389/fcimb.2024.1425367)
Supplement: Supplementary file 4 [file Table_2.docx]

Supplementary Material

**Supplementary Table 2. Potential PGRN-associated proteins of gene microarray**

| **GENE** | **Corrected**  **p-value** | **p-value** | **regulation** | **FC Absolute** | **Fold change** | **Log Fold change** |
| --- | --- | --- | --- | --- | --- | --- |
| GADD45A | 0.0008294 | 0.00011 | up | 3.4193 | 3.4193 | 1.7737 |
| LAMP3 | 0.0007532 | 8.17E-05 | up | 3.17155 | 3.17155 | 1.66519 |
| DCN | 0.0005913 | 3.44E-05 | up | 3.1582 | 3.1582 | 1.6591 |
| VAV3 | 0.0005662 | 2.39E-05 | up | 2.60958 | 2.60958 | 1.38382 |
| PSD3 | 0.0014232 | 0.00053 | up | 2.54995 | 2.54995 | 1.35047 |
| MKNK2 | 0.0008713 | 0.00014 | up | 2.46073 | 2.46073 | 1.29909 |
| LEPR | 0.0004895 | 1.36E-05 | up | 2.34701 | 2.34701 | 1.23082 |
| CTSC | 0.0017936 | 0.00086 | up | 2.21301 | 2.21301 | 1.14601 |
| THBS1 | 0.0015889 | 0.00069 | up | 2.18982 | 2.18982 | 1.13081 |
| FHIT | 0.0016178 | 0.00072 | up | 2.18959 | 2.18959 | 1.13066 |
| CYP1B1 | 0.0005804 | 2.79E-05 | up | 2.14848 | 2.14848 | 1.10332 |
| PLA2G12A | 0.0032152 | 0.00217 | up | 2.13909 | 2.13909 | 1.097 |
| CAV1 | 0.0005913 | 3.50E-05 | up | 2.09375 | 2.09375 | 1.06609 |
| IRS1 | 0.0016156 | 0.00071 | up | 2.03289 | 2.03289 | 1.02353 |
| VTN | 0.0021345 | 0.00119 | down | 2.03103 | -2.03103 | -1.02221 |
| HMOX1 | 0.0017413 | 0.00081 | down | 2.06447 | -2.06447 | -1.04577 |
| SMAD2 | 0.0006497 | 5.07E-05 | down | 2.09848 | -2.09848 | -1.06935 |
| UGT1A10 | 0.0011175 | 0.00029 | down | 2.10122 | -2.10122 | -1.07123 |
| UGT1A8 | 0.0011175 | 0.00029 | down | 2.10122 | -2.10122 | -1.07123 |
| UGT1A7 | 0.0011175 | 0.00029 | down | 2.10122 | -2.10122 | -1.07123 |
| UGT1A6 | 0.0011175 | 0.00029 | down | 2.10122 | -2.10122 | -1.07123 |
| UGT1A5 | 0.0011175 | 0.00029 | down | 2.10122 | -2.10122 | -1.07123 |
| UGT1A9 | 0.0011175 | 0.00029 | down | 2.10122 | -2.10122 | -1.07123 |
| UGT1A4 | 0.0011175 | 0.00029 | down | 2.10122 | -2.10122 | -1.07123 |
| UGT1A1 | 0.0011175 | 0.00029 | down | 2.10122 | -2.10122 | -1.07123 |
| UGT1A3 | 0.0011175 | 0.00029 | down | 2.10122 | -2.10122 | -1.07123 |
| LMO7 | 0.0024488 | 0.00147 | down | 2.17319 | -2.17319 | -1.11982 |
| JUP | 0.0015461 | 0.00066 | down | 2.17432 | -2.17432 | -1.12057 |
| EPB41L3 | 0.0009751 | 0.00021 | down | 2.23946 | -2.23946 | -1.16315 |
| ID3 | 0.0008514 | 0.00012 | down | 2.24179 | -2.24179 | -1.16465 |
| TPM3 | 0.0008133 | 0.0001 | down | 2.25383 | -2.25383 | -1.17238 |
| MAP2K6 | 0.0004895 | 1.32E-05 | down | 2.28963 | -2.28963 | -1.19512 |
| JAK1 | 0.0004882 | 7.13E-06 | down | 2.71242 | -2.71242 | -1.43958 |
| CTSS | 0.0005889 | 3.26E-05 | down | 3.67339 | -3.67339 | -1.87711 |
